# Application of Response Surface Methodology for Modeling of Postweld Heat Treatment Process in a Pressure Vessel Steel ASTM A516 Grade 70

**DOI:** 10.1155/2015/318475

**Published:** 2015-10-15

**Authors:** Prachya Peasura

**Affiliations:** Department of Production Technology Education, Faculty of Industrial Education and Technology, King Mongkut's University of Technology Thonburi, Bangkok 10140, Thailand

## Abstract

This research studied the application of the response surface methodology (RSM) and central composite design (CCD) experiment in mathematical model and optimizes postweld heat treatment (PWHT). The material of study is a pressure vessel steel ASTM A516 grade 70 that is used for gas metal arc welding. PWHT parameters examined in this study included PWHT temperatures and time. The resulting materials were examined using CCD experiment and the RSM to determine the resulting material tensile strength test, observed with optical microscopy and scanning electron microscopy. The experimental results show that using a full quadratic model with the proposed mathematical model is *Y*_TS_ = −285.521 + 15.706*X*_1_ + 2.514*X*_2_ − 0.004*X*_1_^2^ − 0.001*X*_2_^2^ − 0.029*X*_1_ *X*_2_. Tensile strength parameters of PWHT were optimized PWHT time of 5.00 hr and PWHT temperature of 645.75°C. The results show that the PWHT time is the dominant mechanism used to modify the tensile strength compared to the PWHT temperatures. This phenomenon could be explained by the fact that pearlite can contribute to higher tensile strength. Pearlite has an intensity, which results in increased material tensile strength. The research described here can be used as material data on PWHT parameters for an ASTM A516 grade 70 weld.

## 1. Introduction

Pressure vessel steel ASTM A516 Grade 70 is a boiler pressure vessel quality steel that has good weldability and excellent notch toughness and is perfect for moderate and lower temperature services. This material is used extensively by the boiler and pressure vessel fabricators who provide manufacturing support to the petrochemical, oil, and gas industries. The properties and weldability of these steels depend mainly on carbon content. Other elements have only a limited effect. Medium carbon steel, a pronounced change in the weldability of carbon steel, takes place when the carbon content is in 0.30–0.50 percent range. Steel containing about 0.3 percent carbon and relatively low manganese content has good weldability. As the carbon content of the steel is increased, welding procedures must be designed to avoid the formation of large amount of hard martensite in the heat affected zone [1].

The postweld heat treatment (PWTH) or stress-relieving heat treatment is recommended immediately after welding especially with thick section or service condition involving impact or dynamic loading. The martensite transformation and resulting high hardness can lead to cracking in the heat affected zone (HAZ) if the metal cannot yield to relieve welding stress [1, 2].

A central composite design (CCD) contains an imbedded factorial or fractional factorial design with center points which is augmented with a group of “star points” that allow estimation of curvature. If the distance from the center of the design space to a factorial point is ±1 unit for each factor, the distance from the center of the design space to a star point is |*α* | > 1. The precise value of *α* depends on certain properties desired for the design and on the number of factors involved. The CCD is a design widely used for estimating second-order response surfaces. It is perhaps the most popular class of second-order designs. Myers and Montgomery [3] who have studied the CCD in response surface methodology discussed the efficiency of experimental designs and compared the CCD with other designs under *D*-, *A*-, and *E*-optimality criterion. Response surface methodology (RSM) is a collection of mathematical and statistical techniques that are useful for the modeling and analysis of problems in which a response of interest is influenced by several variables and the objective is to optimize the response [4, 5]. The RSM was initially developed and described by Box and coworkers in the study of optimization problems in chemical processing engineering. Mead and Pike [6] and Hill and Hunter [7] conducted earlier work on RSM. This has been used in tool life modeling, surface roughness modeling, and other machining processes. Mital and Mehta [8] developed a predictive surface roughness model for Inconel, using response surface methodology and 2^3^ factorial design of experiment, mathematical models (first-order and second-order) of tool life, surface roughness, and cutting.

In this study, the pressure vessel steel ASTM A516 grade 70 was a risk of cracking to 40% after evaluating the effect of alloying elements (carbon equivalent, CE) in ASTM A516 grade 70. Therefore, the process to prevent cracking can be prevented by using postweld heat treatment. This process consists of PWHT temperature and time gives the most consistent microstructure. This research is the application of response surface methodology (RSM) to find the optimal parameters and the central composite design (CCD) experimental design for a mathematical model to predict the tensile strength [9, 10]. The research has examined the PWHT factors that affect the PWHT time and PWHT temperature. This research can bring about the influence of PWHT and the most appropriate mathematical model as the basis for further ASTM A516 grade 70 weld applications.

## 2. Experimental

### 2.1. Materials and Methods

The research weld material used as the test sample was ASTM A516 grade 70. Plates of research material (6.00 mm thickness) were used for the tests. Details of the material properties are given in Table 1.

The welding samples were gas metal arc welding (GMAW) welded with pressure vessel steel, using a current of 150 Amps direct current electrode negative (DCEN). The welding torch speeds were maintained at 112 mm/sec, and the electrode (ER70s-6) diameter was 0.8 mm [11]. Carbon dioxide (100% CO_2_) in the shielding gas had a flow rate of 12 L/min [12]. After welding, the specimens were PWHT (stress relief). The specimens were treated by PWHT in two factors. The first factor consisted of PWHT time at 5, 10, 15, 20, and 25 hr. The second factor consisted of PWHT temperature examined at 470, 520, 570, and 620°C. Each PWHT condition was conducted randomly, with each condition being tested for a total of three replicates. Welded samples were sectioned transversely to the weld and polished using standard metallographic techniques. The weld specimen were examined and analyzed by the inverted tensile strength test flowing ASTM standard [11]. Polished samples for optical microscope (OM) and scanning electron microscopy (SEM) examination were etched with 2 mL HNO_3_ and 98 mL Methanol [13].

### 2.2. Response Surface Design

Experimental design is widely used for controlling the effects of parameters in many processes. Its usage decreases the number of necessary experiments, which use time and material resources. Furthermore, the analysis performed on the results is easily implemented and experimental errors are minimized. Response surface methodology measures the effect of changes in operating variables and their mutual interactions on the process through experimental design [14, 15]. The response surface methodology is a collection of mathematical and optimization processes that are useful for the modeling and analysis of problems in which a response of interest is influenced by several factors and the aim is to optimize this response. In this study, the CCD was chosen for mathematical modelling of tensile strength. A two-factor second-order model is

(1) y=β0+β1X1+β2X2+β11X112+β22Χ222+β12Χ1Χ2+ε.

Using the central composite design to fit a two-factor second-order model, we have a 2^2^ factorial at ±1, 2(2) axial point and one center point. The matrix, *X*, for this design is given by [16]

(2) $$\begin{array}{c} \left[\begin{array}{cccccc} 1 & -1 & -1 & 1 & 1 & 1\\ 1 & -1 & 1 & 1 & 1 & -1\\ 1 & 1 & -1 & 1 & 1 & -1\\ 1 & 1 & 1 & 1 & 1 & 1\\ 1 & -\alpha & 0 & \alpha^{2} & 0 & 0\\ 1 & \alpha & 0 & \alpha^{2} & 0 & 0\\ 1 & 0 & -\alpha & 0 & \alpha^{2} & 0\\ 1 & 0 & \alpha & 0 & \alpha^{2} & 0\\ 1 & 0 & 0 & 0 & 0 & 0 \end{array}\right],\\ X=\left[\begin{array}{cccccc} N & 0 & 0 & a & a & 0\\ 0 & a & 0 & 0 & 0 & 0\\ 0 & 0 & a & 0 & 0 & 0\\ a & 0 & 0 & b & F & 0\\ a & 0 & 0 & F & b & 0\\ 0 & 0 & 0 & 0 & 0 & F \end{array}\right], \end{array}$$

where *N* is the number of experiment units and *F* is the factorial part of the central composite design. Consider

(3) α=F+2α2,b=F+2α4,

where *α* is the distance from the center of the design and its value is chosen by the experiment for the two-factor central composite design.

The response surface methodology study used CCD; several points are evaluated which increases the chances of detecting the response at which the optimum for a factor occurs. The parameters studied (−*α*, −1, 0, +1, and *α*) in CCD, where levels −1 and +1 represent the low and high values, −*α* and *α* indicate the low and high extreme values, and 0 is the center value of each parameter [17, 18], were shown in Tables 2, 3, and 4.

## 3. Result and Discussion

### 3.1. Response Surface Model of Tensile Strength

A model was used to determine the tensile strength and to predict the optimization of PWHT temperature and PWHT time. Statistical analysis was used to obtain the results and conclusions of the trial through analyzing the variability in the experimental CCD of model analysis to determine the coefficient of determination *R* ^2^ Adj. and the lack-of-fit of the experimental data [19, 20]. Independent variables and their levels for the CCD used in this study are shown in Tables 5 and 6.

From the estimated regression listed in Table 5, it was found that the *P* values of the PWHT temperature (*P* value = 0.000) and PWHT time (*P* value = 0.000) with the *P* value > 0.05 indicate that all three terms are important. Data analysis of the full quadratic equations, in terms of *R* ^2^ = 87.90% and *R* ^2^ Adj. = 85.89%, satisfies coefficients of the *P* value of regression, which is 0 < *α*; thus we reject the null hypothesis. The functions in terms of full quadratic regression are linear and the least variable regression of tensile strength will significantly affect the modeled mathematical equations of the *P* value of lack-of-fit equal to 0.185 which is >*α*. The terms of the full quadratic equation, which are sufficient, are shown in Table 6.

The model is a mathematical model from Table 7. Consider

(4) yTS=−285.521+15.706X1+2.514X2−0.004X12−0.001X22−0.029X1X2.

**Figure 1:**
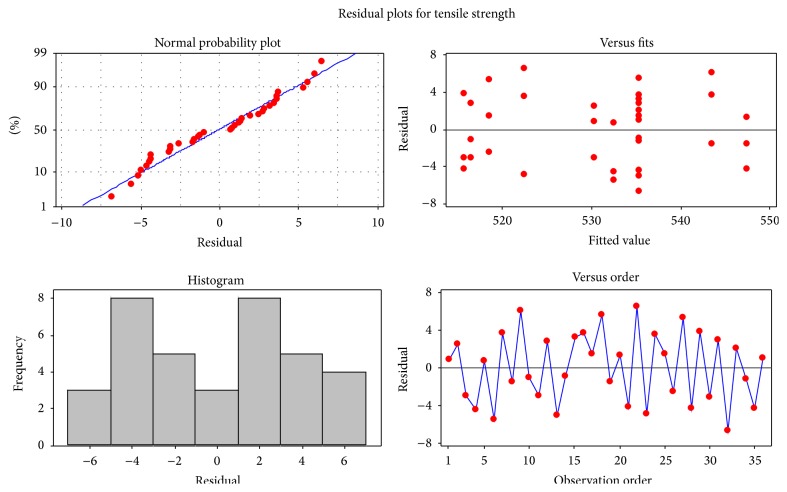
Residual plot for tensile strength from mathematical model checking.

The model adequacy check was performed and validated by experimental models. The hypothesis is that the pattern of the residuals obtained from the experimental data adheres to the principle *ε*_*ij*_ ~ NID (0, *σ* ^2^). Residuals are assumed to be independent and normally distributed. The mean 0 and *σ* ^2^ is near stable to the experimental data. Accurate and reliable monitoring is possible by the stated assumptions shown in Figure 1.

**Figure 2:**
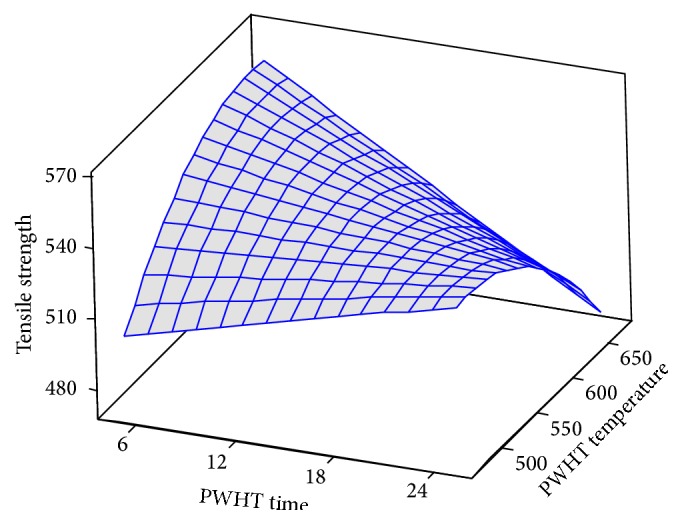
Response surface of tensile strength for PWHT temperature and time.

Response surface methodology results from Figure 2 show that the experimental tensile strength decreases with PWHT temperature. Variation of the tensile strength is reduced in relation to PWHT temperature. Therefore, maximum values for tensile strength are obtained at minimum PWHT time and medium PWHT temperature. It is noted that, for the focus inside piece, tensile strength is higher than that for the focus to the work piece surface of the sample. This is explained by decreasing the PWHT temperature at the sample for the PWHT factor focus within the sample.

**Figure 3:**
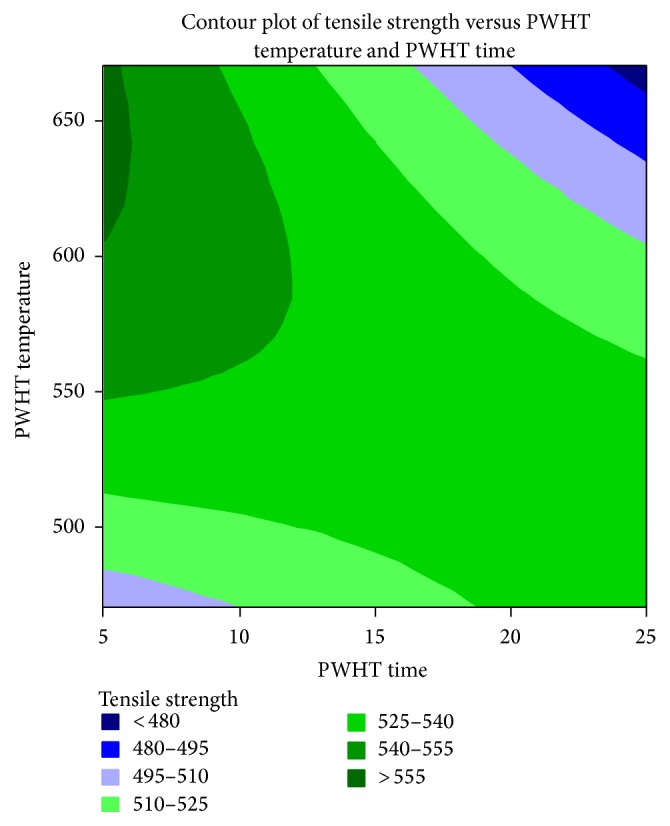
Response surface plots of PWHT time and PWHT temperature for tensile strength.

Figure 3 shows the 2D response surface plot for tensile strength. The optimum conditions as stated by further numerical analysis of the responses with design expert software reveal that the most influencing variable is the PWHT parameter. The response surface plot shows the optimized tensile strength at low PWHT time and medium PWHT temperature.

**Figure 4:**
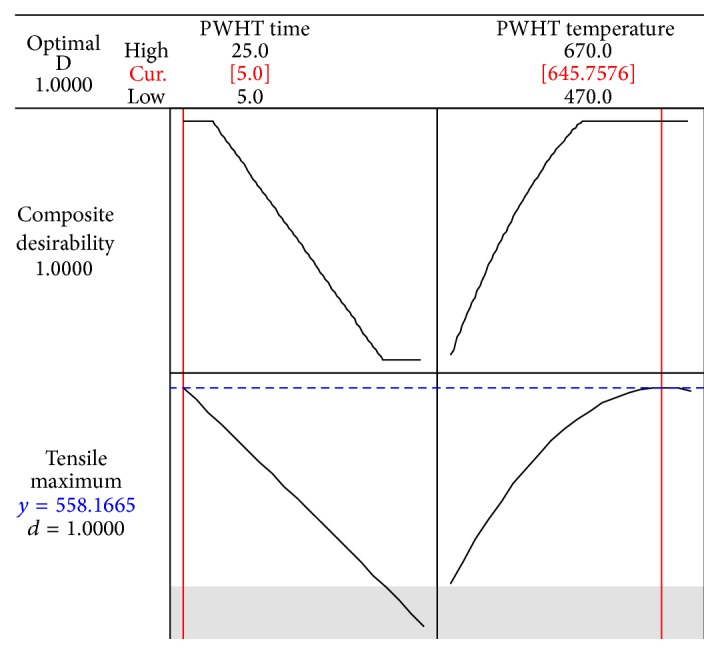
Response optimization of tensile strength.

The analysis was conducted to determine the appropriate level of the three factors that result in tensile strength. The graph of the main factors influencing tensile strength using equation *y*_TS_ = −285.521 + 15.706*X*_1_ + 2.514*X*_2_ − 0.004*X*_1_^2^ − 0.001*X*_2_^2^ − 0.029*X*_1_ *X*_2_ with restrictions 5 ≤ *X*_1_ ≤ 25, 470 ≤ *X*_2_ ≤ 670 and *X*_1_, *X*_2_ > 0 is shown in Figure 4.

The parameters of PWHT optimization are as follows:

1. PWHT time of 5.00 hr.
2. PWHT temperature of 645.75°C.

Tensile strength is 588.16 MPa.

### 3.2. Validation Model Experiments

This confirmation experiment was performed for validation. For this confirmation experiment, the tensile strength value and PWHT parameters were selected from the given specified range and five experiments were performed for tensile strength. The experimental values obtained from confirmation testing were compared with the mathematical model for predicted tensile strength. It is observed from the results that the compared error is 0.92%, as shown in Table 8.

### 3.3. Microstructure Analysis

Analysis was conducted to determine how microstructure analysis in the HAZ microstructure affects the weld specimens. A variety of characterization techniques were used for the current study, including optical microscopy and scanning electron microscope. The OM and SEM analyses of the microstructure are needed for analysis of each PWHT factor. SEM uses a focused beam of high-energy electrons to generate a variety of signals at the surface of a solid sample and was used after the test specimens were prepared according to the instructions of the ASTM standard test.

**Figure 5:**
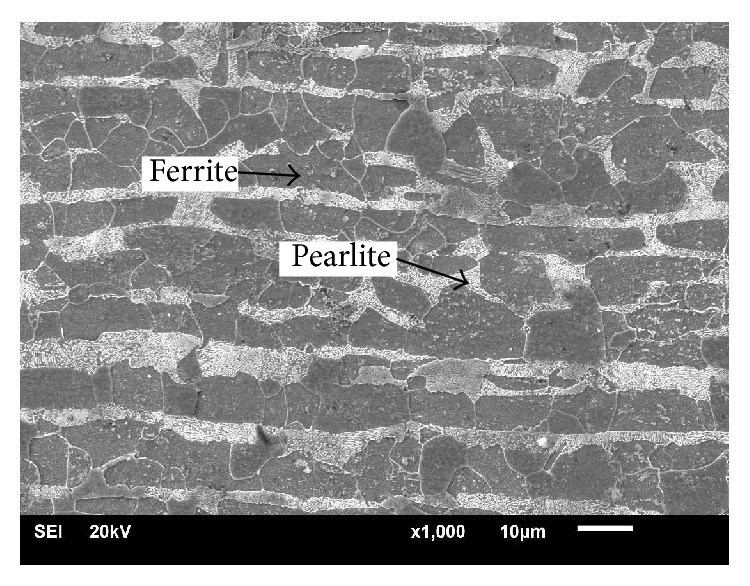
Microstructure of as-received material ASTM A516 grade 70.

The ASTM A516 grade 70 microstructure of the as-received material is shown in Figure 5; the SEM microstructure was a ferrite phase matrix and pearlite phases, and there was significant pearlite banding in the rolling plane [21, 22].

**Figure 6:**
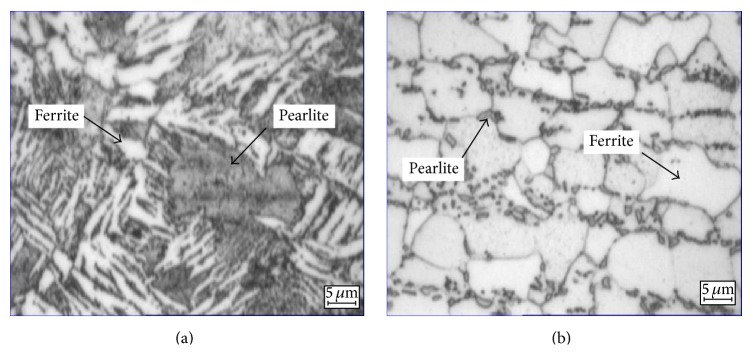
HAZ microstructure of sample in a PWHT with (a) PWHT temperature of 645°C and PWHT time of 5 hr and (b) PWHT temperature of 670°C and PWHT time of 25 hr.

The HAZ micrograph in Figure 6 compares the ferrite and pearlite phases in the specimens.  Figure 6(a) also shows the microstructure for the specimen with a PWHT treatment at 645°C for 5 hr, which shows an increase in density of the pearlite which results in a maximum material hardness. At a PWHT temperature of 670°C and a PWHT time of 25 hr, overstress relief condition in the HAZ results in coarsening grain size which was caused by the over-PWHT time and temperature for coarsening grain size and can contribute to the lower hardness [23, 24].

## 4. Conclusions

In this study, the application of RSM and CCD to tensile strength tests was discussed. The three-level two-factor CCD experimental design was applied in the study. Variables of the model investigated in this study were PWHT time and PWHT temperature. The results from this study are summarized as follows.

(1) Design of experiments was used to determine suitable PWHT temperatures and times. The tensile strength parameters of PWHT are an optimized PWHT time of 5.00 hr and PWHT temperature of 645.75°C.
(2) Predicted values of tensile strength obtained using a mathematical model were in agreement with experimental values, which follow
  (5) yTS=−285.521+15.706X1+2.514X2−0.004X12−0.001X22−0.029X1X2.
(3) The PWHT time is the dominant mechanism used to modify the tensile strength compared to the PWHT temperatures. This phenomenon could be explained by the fact that pearlite can contribute to higher tensile strength. The pearlite has an intensity, which results in increased material tensile strength.

## Figures and Tables

**Table 1:** Chemical composition of ASTM A516 grade 70.

Chemical element	C	Si	Mn	P	S
% [wt]	0.28	0.13	0.79	0.035	0.04

**Table 2:** Experimental factors for response surface methodology.

Parameters	Symbols	Units	Levels				
			−*α*	−1	0	1	*α*
PWHT time	*X*_1_	hr	5	10	15	20	25
PWHT temperature	*X*_2_	°C	470	520	570	620	670

**Table 3:** CCD design with 3 center points for the tensile strength test.

Block	*X*_1_	*X*_2_
1	−1	−1
1	1	−1
1	−1	1
1	1	1
1	0	0
1	0	0
2	−*α*	0
2	*α*	0
2	0	−*α*
2	0	*α*
2	0	0
2	0	0

**Table 4:** Matrix of PWTH parameters and response factor in tensile strength.

Std. order	Run order	PWHT time	PWHT temperature	Tensile strength (MPa)
1	9	10	520	531.10
2	12	10	520	532.80
3	20	10	520	527.20
4	1	20	520	528.00
5	5	20	520	533.30
6	18	20	520	527.00
7	10	10	620	547.30
8	25	10	620	542.00
9	2	10	620	549.70
10	29	20	620	515.20
11	19	20	620	513.30
12	32	20	620	519.20
13	36	15	570	530.25
14	11	15	570	534.44
15	4	15	570	538.60
16	27	15	570	539.10
17	7	15	570	536.80
18	15	15	570	541.00
19	17	5	570	545.88
20	22	5	570	548.71
21	35	5	570	543.13
22	26	25	570	529.00
23	14	25	570	517.50
24	31	25	570	526.00
25	3	15	470	520.00
26	28	15	470	516.00
27	23	15	470	523.90
28	8	15	670	511.32
29	30	15	670	519.48
30	13	15	670	512.50
31	33	15	570	538.25
32	6	15	570	528.62
33	34	15	570	537.44
34	21	15	570	534.10
35	24	15	570	531.00
36	16	15	570	536.40

**Table 5:** Estimated regression coefficients for tensile strength.

Term	Coef.	SE coef.	*T*	*P*
Constant	535.400	1.064	503.171	0.000
PWHT time	−12.474	1.346	−9.268	0.000
PWHT temp.	−1.439	1.346	−1.069	0.294
PWHT time *∗* PWHT time	−0.463	2.019	−0.229	0.820
PWHT temp. *∗* PWHT temp.	−18.300	2.019	−9.064	0.000
PWHT time *∗* PWHT temp.	−29.500	4.662	−6.327	0.000

*S* = 4.037795.134; *R* ^2^ = 87.90%; *R* ^2^(adj.) = 85.89%.

**Table 6:** Analysis of variance for tensile strength.

Source	DF	Seq. SS	Adj. SS	Adj. MS	*F*	*P*
Regression	5	3554.36	3554.36	710.87	43.60	0.000
Linear	2	1419.14	1419.14	709.57	43.52	0.000
Square	2	1482.53	1482.53	741.27	45.47	0.000
Interaction	1	652.69	652.69	652.69	40.03	0.000
Residual error	30	489.11	489.11	16.30		
Lack-of-fit	3	78.79	78.79	26.26	1.73	0.185
Pure error	27	410.32	410.32	15.20		
Total	35	4043.47				

**Table 7:** Estimated regression coefficients for tensile strength test.

Term	Coef.	Symbol
Constant	−285.521	—
PWHT time	15.7066	*X*_1_
PWHT temperature	2.51431	*X*_2_
PWHT time *∗* PWHT time	−0.00463333	*X*_1_^2^
PWHT temp. *∗* PWHT temp.	−0.00183000	*X*_2_^2^
PWHT time *∗* PWHT temp.	−0.0295000	*X*_1_ *X*_2_

**Table 8:** Validation model experiments: comparisons with the predicted values.

Experiments number	PWHT temperature °C	PWHT time (hr)	Tensile strength (MPa)		Error %
			Experiments	Predicted	
1	645.75	5.00	580	588	−1.37
2	645.75	5.00	594	588	1.01
3	645.75	5.00	585	588	−0.52
4	645.75	5.00	592	588	0.68
5	645.75	5.00	582	588	−1.02
